# Facile Enhancement of Mechanical Interfacial Strength of Recycled Carbon Fiber Web-Reinforced Polypropylene Composites via a Single-Step Silane Modification Process

**DOI:** 10.3390/polym17040483

**Published:** 2025-02-12

**Authors:** Yeo-Jun Song, Dong-Kyu Kim, Woong Han, Sun-Ho Choi, Dong-Chul Chung, Kwan-Woo Kim, Byung-Joo Kim

**Affiliations:** 1 Industrialization Division, Korea Carbon Industry Promotion Agency, Jeonju 54852, Republic of Korea; sgh3444@naver.com (Y.-J.S.); kdg9141@kcarbon.or.kr (D.-K.K.); shareyi@kcarbon.or.kr (W.H.); 2 Department of Carbon Materials and Fiber Engineering, Jeonbuk University, Jeonju 54896, Republic of Korea; 3 Department of Carbon Convergence Engineering, Jeonju University, Jeonju 55069, Republic of Korea; rokmwinning2014@gmail.com; 4 Department of Organic Materials and Textile Engineering, Jeonbuk National University, Jeonju 54896, Republic of Korea; 5 Department of Materials Science and Chemical Engineering, Jeonju University, Jeonju 55069, Republic of Korea

**Keywords:** recycled carbon fiber, wet laid, silane treatment, composites

## Abstract

In this study, a surface treatment process was introduced into the conventional dispersion process for preparing wet-laid nonwoven fabrics to improve their properties, using recycled carbon fibers (rCFs). The conventional binder solution was replaced with a solution containing different amounts of silane, and the changes in the fiber properties of the prepared nonwoven fabrics were examined after the addition of modified rCFs and polypropylene. FE-SEM analysis confirmed that a silane layer was formed on the rCF surface due to the formation of a siloxane network. FT-IR and XPS analyses further confirmed the presence of siloxane bonds and chemical modification of the rCF surface. When an optimal amount of silane content was used, the mechanical strength increased by 64% compared to untreated rCFs, owing to the improved molecular chain entanglement within the matrix. Our findings indicate that the simultaneous use of dispersion and a surface treatment can produce composites with excellent mechanical properties and improved processing and surface properties; thus, this method can be used to help upcycle rCFs, thereby expanding their applications.

## 1. Introduction

Carbon fiber-reinforced plastics (CFRPs) have attracted considerable attention as ultra-lightweight advanced composites [1,2]. Owing to their advantageous properties, such as high specific strength, corrosion resistance, and heat resistance, they are widely used in the automotive, marine, sporting goods, construction, and aerospace industries [3,4,5,6]. The global demand for CFRPs has increased by approximately 12.5% over the past 20 years; however, because of the high demand and their long lifecycle, the amount of generated CFRP waste will reach approximately 263,000 tons by 2030 [7,8]. Because CFRP waste does not naturally decompose easily, it is typically disposed of via incineration, followed by landfilling, which can have harmful long-term effects on the environment [9,10]. Recently, as global awareness of environmental pollution continues to rise, European Union countries have started to ban the use of mixed waste landfills [11,12]. Therefore, to reduce carbon emissions and waste generation, it would be efficient to recover high-quality carbon fibers (CFs) from CFRP waste and reuse them to produce valuable new materials. This approach can reduce the reliance on virgin CFs, as well as reduce carbon emissions and the related costs. Therefore, based on the important environmental and economic benefits involved, research on the conversion of CFRP waste into high-value materials is required.

Typically, CFs recycled from CFRP waste are recovered in the form of short fibers via grinding, shredding, and cutting, which limits their application. To solve these problems and expand the fields of application of recycled carbon fibers (rCFs), they are being reused as a composite material together with thermoplastic polymers. Thermoplastic polymers have recently attracted attention as important materials in CFRP recycling research due to their various advantageous properties, including high production efficiency, excellent impact resistance, and recyclability. Among various thermoplastic polymers, the application range of polypropylene (PP) in regard to various fields is expanding due to its high mechanical strength, low density, excellent chemical resistance, and low price. To expand the application of rCFs into more diverse fields, nonwoven fabrics are being produced by mixing them with thermoplastic polymers, using different methods, such as wet-laid [13,14,15], air-laid [16,17,18], and carding [19,20,21] methods, to broaden its applications. Among these methods, the wet-laid method can be used on nonwoven fabrics, by uniformly dispersing fibers in a solution containing a binder and then filtering them through a fine mesh, which is simpler than other methods and produces nonwoven fabrics with a uniform fiber distribution. This method is widely used in the paper and textile industries owing to the benefits it offers, such as high productivity, fiber orientation control, fiber blend utilization, and low production costs [22,23,24]. However, owing to the nonpolar and chemically inert nature of rCFs and PP, even if CFRP is manufactured as a wet-laid nonwoven fabric without surface treatment, its mechanical properties may be poor due to the weak interfacial bonding within the matrix [25,26]. In particular, improving the interfacial bonding strength of rCFs with polypropylene, among the different thermoplastic resins available, is considered a difficult problem. Therefore, the surface treatment of rCFs is essential for enhancing the interfacial bonding of CFRP composites [27].

The application of a surface treatment is an effective method to improve the mechanical properties of CFRP, by addressing the weak interfacial adhesion between the fibers and the matrix [28]. Various methods have been applied in regard to surface treatment, including chemical [29,30], electrochemical [31,32,33], sizing [34,35,36], thermal [37,38], plasma [39,40,41], and coupling treatments [42,43,44,45]. Among the various surface treatment methods, silane treatment of the surface is commonly used for textiles and has been widely reported in various fields [46]. Silane coupling agents have double functional groups that can increase the wettability of the CF surface after silane treatment and form strong covalent bonds between the CFs and the matrix to improve compatibility. In addition, interpenetrating polymer networks (IPNs), formed by the physical entanglement between the siloxane network and the polymer matrix, can enhance the interfacial binding between the silane and the matrix [47,48,49].

In this study, a conventional binder solution, used to connect the fibers during the dispersion process for preparing wet-laid nonwoven fabric, was replaced with a silane solution for simultaneous surface treatment and binding. Furthermore, rCF/polypropylene (PP) wet-laid nonwoven fabrics were fabricated with different amounts of silane content to examine the effect of silane content on their mechanical strength.

## 2. Materials and Methods

### 2.1. Materials

The rCF wet-laid nonwoven fabric prepared in this study was composed of rCFs (6 mm, Catack-H, Hwaseong, Republic of Korea) as the reinforcement and PP fibers (6 mm, Nycontech, Asan, Republic of Korea) as the matrix. Moreover, 3-aminoproplytriethoxysilane (APTES, 98%, Sigma-Aldrich, St. Louis, MO, USA) was used for the rCF surface treatment and as a binding additive.

### 2.2. Preparation of Silane-Treated Nonwoven Fabrics

**Figure 1:**
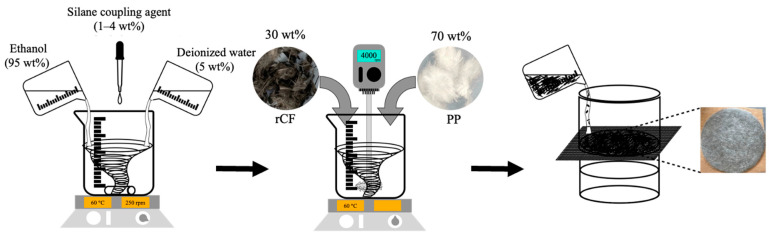
Silane treatment process and rCF/PP nonwoven fabric production.

Ethanol and distilled water were added to a beaker, with a 95:5 wt% ratio, and stirred for 15 min at 60 °C. Then, 1–4 wt% of the silane coupling agent was added to the mixed aqueous solution and stirred at 60 °C for an additional 15 min to prepare the silane solution. The silane solution was kept at 60 °C. The total weight ratio of the rCF and PP was fixed at 30:70 wt%, considering the balance between strength and processability, and the rCF content was selected as 30 wt%, and then mixed [50,51,52]. After incorporation, the samples were dispersed using a homogenizer at 4000 rpm, for 20 min. The dispersed fiber mixture was filtered through an iron mesh, using in-house wet-laid equipment to produce the nonwoven fabrics, which were dried for 12 h in an oven at 80 °C. The final wet weight of the nonwoven fabric was 200 g/m^2^, and the fabric was used to manufacture the composites. A schematic diagram of the process is shown in Figure 1.

### 2.3. Fabrication of Nonwoven Composites via Hot-Press Molding

**Figure 2:**
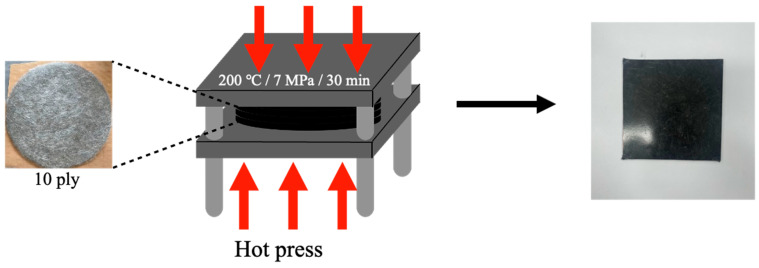
Fabrication of rCF/PP nonwoven composites.

**Figure 3:**
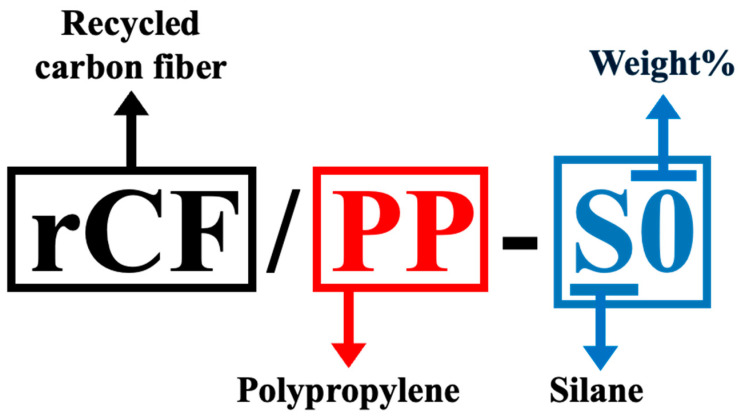
Nomenclature for recycled carbon fiber based on silane treatment.

The rCF/PP wet-laid nonwoven composite was fabricated via hot pressing, as shown in Figure 2. The prepared rCF/PP wet-laid nonwoven fabric was laminated into 10 layers (i.e., 10 ply) and placed in a mold. The temperature was maintained at 200 °C for 30 min at 7 MPa to ensure full contact and heat transfer. The sample was allowed to cool naturally to 40 °C, before demolding to produce the composite. The sample names and nomenclature for the different production conditions are shown in Table 1 and Figure 3.

### 2.4. Characterization

The surface morphologies of the untreated and silane-treated rCFs were examined via field emission scanning electron microscopy (FE-SEM, SUPRA40VP, Carl Zeiss, Oberkochen, Germany). To minimize any charging effects during the analysis, the rCFs were attached to the holder using carbon tape and coated with Pt 100 s before the observation. The base pressure in the FE-SEM chamber was approximately 2.16 × 10^−4^ Pa, and the accelerating voltage was set to 2.0 kV. The functional groups on the rCF surface were identified and analyzed using Fourier transform infrared (FT-IR) spectroscopy (Nicolet iS10, Thermo Fisher Scientific, Waltham, MA, USA). The FT-IR samples were prepared by grinding and pelletizing the untreated and silane-treated rCFs and KBr together, and were measured between 4000 and 500 cm^−1^. To confirm the surface chemistry of the rCFs, X-ray photoelectron spectroscopy (XPS; Nexsa XPS System, Thermo Fisher Scientific, Waltham, MA, USA) measurements were carried out using monochromatic Al-*K*_a_ (1486.6 eV) X-rays. During the XPS measurements, the pass energy was fixed at 50 eV, and the base pressure in the chamber was 2.6 × 10^−5^ Pa.

The mechanical properties of the rCF/PP composites were determined via a three-point bending test, according to the ASTM D790 standard [53], using a universal testing machine (ST-1001, Salt, Namyangju, Republic of Korea). The rCF/PP composite specimen size was 60 mm (length) × 12.7 mm (width) × 2.4 mm (thickness), the span distance was 32 mm, and the downward speed of the pressure head was measured at 1 mm/min. Eight rCF/PP composites were tested.

To calculate the void content, the actual densities of the composites were measured using the Archimedes test (Mettler Toledo XS204, Hogentogler, Columbia, MD, USA). According to the ASTM D2734 standard, porosity (Xv) can be calculated using Equation (1):

(1) $$X_{v}=100\%\times \left(p_{t}-p_{a}\right)/p_{t},$$

where $X_{v}$ is the porosity, $p_{t}$ is the theoretical density of the composite, and $p_{a}$ is the actual density.

## 3. Results and Discussion

### 3.1. Characteristics of the Functionalized rCF Surface

**Figure 4:**
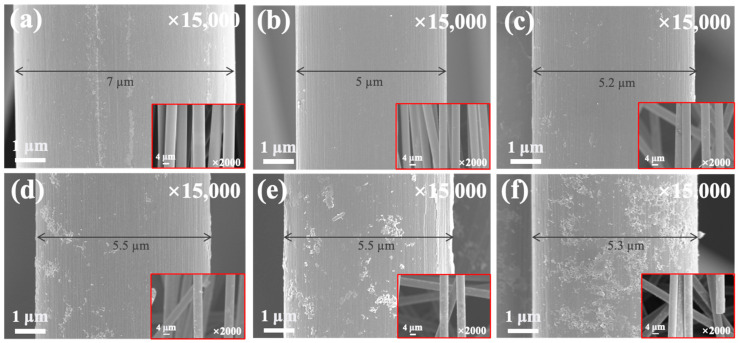
FE-SEM images of the untreated and silane-treated rCFs with various amounts of silane content: (**a**) rCFs, (**b**) rCF-S0, (**c**) rCF-S1, (**d**) rCF-S2, (**e**) rCF-S3, and (**f**) rCF-S4.

The FE-SEM images of the surface morphologies of the untreated and treated rCFs are shown in Figure 4. Figure 4a shows the surface morphology of the untreated rCFs, confirming that the matrix residues that were not degraded during the recycling process remained on the surface of the rCFs. Figure 4c–f presents the surface morphologies of rCFs after silane treatment. As shown, an island-shaped silane layer was formed on the rCF surface. Additionally, the distribution of silane layers on the rCF surface increased, in the form of snowflakes, as the silane content increased. The increasing amount of silane content likely enhanced the mechanical interlocking between the fibers and the matrix owing to the increased roughness of the rCF surface and increased surface area, thereby enhancing the interfacial bonding. A comparison of Figure 4a,b–f reveals that the diameter of the rCFs was reduced, indicating the removal of defects, amorphous structures, and residues from the outermost surface of the rCFs. Such a finding was attributed to the physical influence of the homogenizer during the dispersion process, yielding a uniform rCF surface [48].

**Figure 5:**
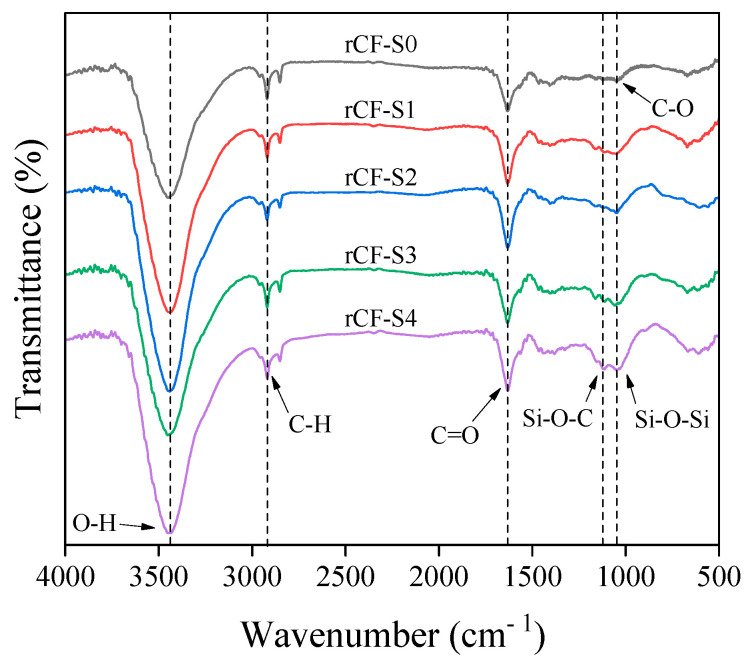
FT-IR spectra of the untreated rCFs and rCFs treated with various amounts of silane content.

**Figure 6:**
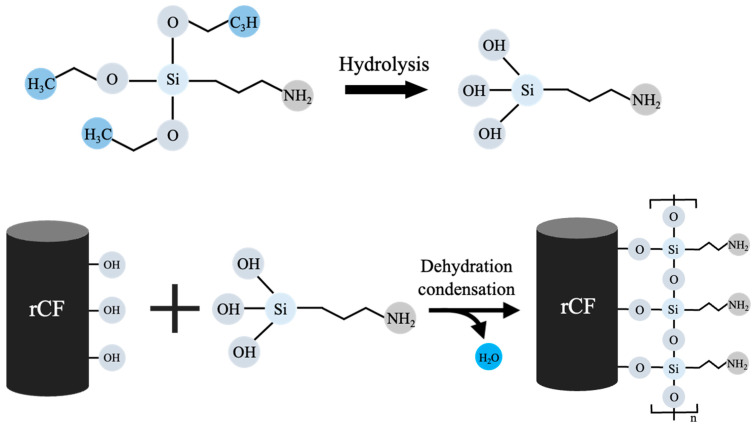
Mechanism of the silane coupling treatment on the rCF surface.

The changes in the functional groups of the rCFs after surface treatment were determined via FT-IR spectroscopy, and the results are shown in Figure 5. The spectrum of the untreated rCFs contained bands corresponding to the stretching vibrations of the O-H, C=O, and C-O groups at 3440, 1630, and 1050 cm^−1^, respectively [47]. For the rCFs treated with various amounts of silane content, new Si-O-C and Si-O-Si peaks appeared in the spectra between 1120 and 1035 cm^−1^ [47]. This change can be explained as follows: the terminal Si-OR group of the silane coupling agent was hydrolyzed using a mixed aqueous solution of water and ethanol to form a more reactive silanol (Si-OH) group, which activated the silane. Subsequently, the hydroxyl groups and Si-OH on the rCF surface bonded to form a siloxane network structure in the form of Si-O-Si, via dehydration and condensation reactions [54]. This siloxane network formation likely improved the interfacial bonding between the fibers and the matrix, enhancing the physical properties of the composites. The mechanism of silane treatment in terms of the rCF surface is shown in Figure 6. 

**Figure 7:**
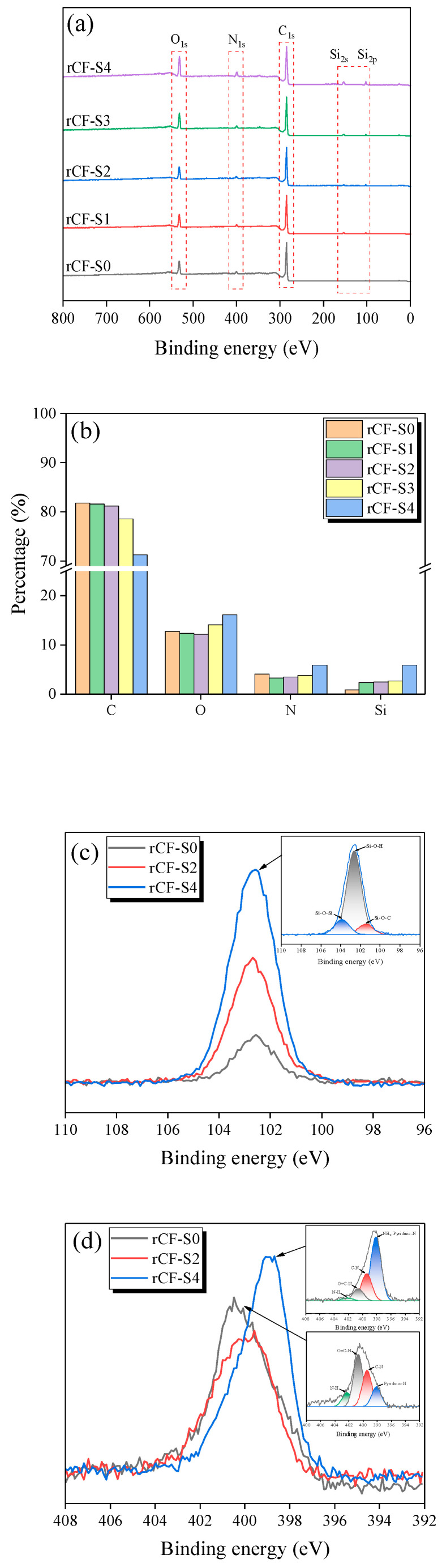
XPS spectra of the untreated and silanized rCFs: (**a**) XPS survey spectra, (**b**) surface elemental contents of the untreated and silane-treated rCFs, (**c**) fitting curves of the Si2p peaks for the rCF-S0 and rCF-S4 samples, and (**d**) fitting curves of the N_1s_ peaks for the rCF-S0 and rCF-S4 samples.

To further analyze the changes in the chemical elements and functional groups on the rCF surface after silane treatment, XPS measurements were performed (Figure 7). The spectra of all the rCF surfaces contained peaks corresponding to C, O, N, and Si. Notably, the rCF-S0 surface exhibited the highest elemental C content (82.13%), and the Si content increased with the increase in silane content; briefly, the N, O, and Si contents of the rCF-S4 sample treated with the highest amount of silane content increased by 26.23%, 44.71%, and 603.53%, respectively, compared with that of the rCF-S0 sample. These results indicate that a siloxane network was successfully formed on the rCF surface.

The chemical bonding of the untreated rCF and silanized rCF surfaces was analyzed by fitting the N_1s_ and Si_2p_ peaks in the XPS spectra (Figure 7c,d). The high-resolution Si_2p_ spectra of the silane-treated rCFs were deconvoluted into three peaks, corresponding to Si-O-H (102.2 eV), Si-O-C (103.2 eV), and Si-O-Si (101.4 eV) [47]. It is considered that the intensity of these peaks increased as the O-H functional group on the rCF surface bonded with silanol and formed a siloxane network via a condensation reaction [54]. The high-resolution N_1s_ spectrum of rCF-S0 was deconvoluted into four peaks, corresponding to N-H (402.3 eV), O=C–N (400.6 eV), C-N (399.5 eV), and pyridine (398 eV) [55]. The fitted N_1s_ spectra of the rCFs after silane treatment contained a peak corresponding to NH_2_ (398 eV), because of the NH_2_ functional groups located at the siloxane network terminals. Abundant NH_2_ functional groups can increase the number of reaction sites between rCFs and the matrix, strengthening the fiber–matrix bond [55]. The XPS results confirm the successful introduction of the siloxane network onto the rCF surface, which is consistent with the FT-IR characterization results.

### 3.2. Void Content and Fiber Density

The density and porosity of the untreated and silane-treated rCF/PP composites are presented in Table 2, revealing a reduction in porosity after the silane treatment. The untreated rCF/PP composite has the highest porosity (24%), indicating poor fiber–matrix interfacial interactions. However, as the silane content increased from 1 to 4 wt%, the porosity decreased from 22% to 16%, which can be attributed to the strong interfacial bonding between the silane-treated rCFs and the PP matrix.

### 3.3. Mechanical Interfacial Properties

**Figure 8:**
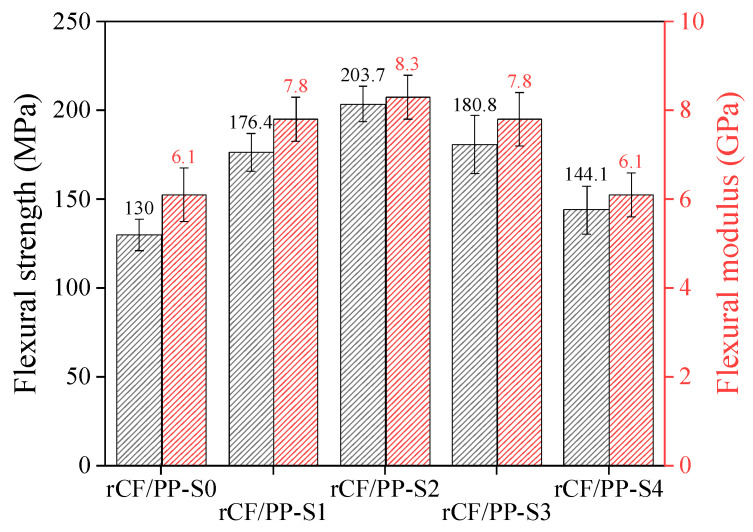
Flexural strength and modulus of the untreated and silane-treated rCF/PP composites.

The flexural strength of the untreated and silane-treated rCF/PP composites is presented in Figure 8. The rCF/PP-S2 (silane content of 2 wt%) sample exhibited an increase in flexural strength of up to 64% compared with that of the rCF/PP-S0 sample, along with an approximate 36% increase in the flexural modulus. However, when the silane treatment content was increased beyond a certain level, the flexural strength decreased. In the case of the rCF/PP-S4 sample, the flexural strength decreased by up to 38% compared with that of the rCF/PP-S2 sample, and the flexural modulus decreased by approximately 27%. This result can be attributed to the increase in flexural strength as the siloxane network and thermoplastic matrix were produced on the surface of the rCFs during silane treatment, forming an IPN, owing to the physical entanglement of the molecular chains, which resulted in stable and strong interfacial bonding [47]. However, excessive use of a silane coupling agent forms an excessive silane layer on the rCF surface. This increases the amount of unstable bonds and causes the concentration of interbond stress, which is gradually transmitted to the interface between the rCFs and the silane layer, leading to easy debonding [56]. Therefore, the flexural strength decreased when the composite was treated with a silane content ≥2 wt%. Jinshui et al. [56] reported that with an increase in the concentration of the silane coupling agent, the excess silane layer has a lubricating effect on the composite’s interphase, which reduces the flexural strength. The results in the present study indicate that the optimal silane content in terms of silane-treated rCF/PP composites is 2 wt%.

### 3.4. SEM Observation of the Fracture Region

**Figure 9:**
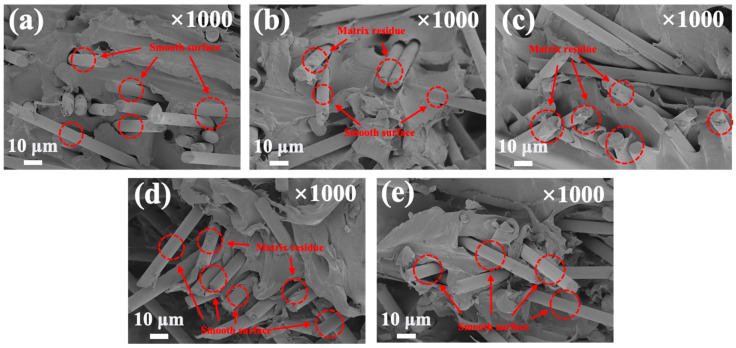
FE-SEM images of the fractured surfaces of untreated and silane-treated rCF/PP composites: (**a**) rCF/PP-S0, (**b**) rCF/PP-S1, (**c**) rCF/PP-S2, (**d**) rCF/PP-S3, and (**e**) rCF/PP-S4.

After the flexural strength tests, the fracture surfaces of the untreated and silane-treated rCF/PP composites were examined via FE-SEM, and the micrographs are shown in Figure 9. Regarding the rCF/PP-S0 sample, the rCF surface was relatively smooth and clean, and the amount of matrix residue attached to the surface was small. Conversely, in regard to the rCF/PP-S2 sample, the rCFs were tightly bound with the matrix and a larger amount of matrix residue was observed on the rCF surface compared with the rCF/PP-S0 sample (Figure 9c). This can be attributed to the siloxane network formed on the rCF surface, exhibiting a high degree of interfacial bonding after the addition of PP and the formation of an IPN, resulting in matrix residues on the rCF surface, even after fracturing. However, in regard to the rCF/PP-S3 and rCF/PP-S4 samples, the rCF surface became smoother, and the amount of matrix residue attached to the surface decreased (Figure 9d,e). These results can be attributed to the reduction in the interfacial bonding force between the rCFs and the matrix, as confirmed by the flexural strength measurements, and the reduction in the amount of matrix residue attached to the rCF surface after debonding.

**Figure 10:**
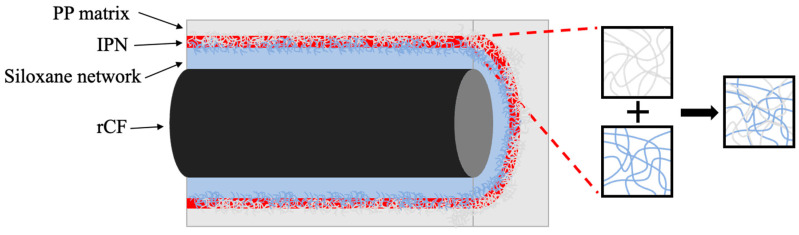
Mechanism of the interface enhancement in the rCF/PP composites.

The mechanism of bond strengthening in the rCF/PP composites caused by the silane treatment of the rCF surface is shown in Figure 10. During silane treatment, the hydroxyl groups on the rCF surface and silanol are covalently bonded to form a siloxane network; subsequently, an IPN is formed through the physical entanglement of the PP matrix and molecular chains, which likely increases the flexural strength by improving the interfacial bonding between the rCFs and the PP matrix [44]. In conclusion, the chemical and physical interactions between the rCFs and the PP matrix can significantly increase the interfacial bonding force, enhancing the properties of the composite.

## 4. Conclusions

In this study, to improve the wet-laid process and enhance the interfacial bonding between rCFs and the matrix, we prepared rCF wet-laid nonwoven fabrics. By replacing the binder-containing solution with a silane solution, we simplified the preparation process, integrating dispersion, surface treatment, and binding. Surface analysis indicated that the distribution of island-shaped siloxane networks on the rCF surface increased with the increase in silane content. In addition, FT-IR and XPS analyses identified the Si-O-C and Si-O-Si functional groups, and the increase in the intensity of the N_1s_ peak confirmed the successful introduction of the siloxane network on the rCF surface. Mechanical property-related measurements indicated that the flexural strength of the silane-treated rCFs was 64% higher than that of untreated rCFs. Such a finding was attributed to the hydroxyl groups on the rCF surface forming covalent bonds with silanol groups and the siloxane network forming an IPN via molecular chain entanglement with the matrix, which increased the flexural strength. However, the flexural strength decreased when the silane treatment content was increased beyond 2 wt%. This decrease can be attributed to the formation of an excessive silane layer on the rCF surface, which reduced the specific surface area and increased the number of unstable bonds, degrading the physical properties of the composite because of the stress concentration between the bonds and the destruction of the interface. In conclusion, by replacing the solution containing the binder and rCFs in the wet-laid process with an optimal silane solution, nonwoven fabrics with enhanced uniformity and surface properties can be produced, resulting in composites with excellent mechanical properties. This demonstrates their potential applicability in fields such as future mobility and sports equipment industries, aligning with current industrial trends that emphasize environmental issues and the use of lightweight materials, based on enhanced interfacial bonding and excellent mechanical properties.

## Figures and Tables

**Table 1:** Sample names and treatment conditions.

Fiber		Composite				
Sample	Silane Content (wt%)	Sample	P (MPa)	T (°C)	Holding Time (min)	Natural Cooling (°C)
rCF-S0	-	rCF/PP-S0	7	200	30	40
rCF-S1	1	rCF/PP-S1				
rCF-S2	2	rCF/PP-S2				
rCF-S3	3	rCF/PP-S3				
rCF-S4	4	rCF/PP-S4				

**Table 2:** Porosity and density of the untreated and silane-treated Flexural strength and modulus of the untreated and silane-treated rCF/PP composites.

Sample	Theoretical Density (g/cm^3^)	Density (g/cm^3^)	Void Content (%)
rCF/PP-S0	1.054	0.790	24.739
rCF/PP-S1		0.812	22.699
rCF/PP-S2		0.839	20.124
rCF/PP-S3		0.852	18.830
rCF/PP-S4		0.882	16.027

## Data Availability

The original contributions presented in the study are included in the article; further inquiries can be directed to the corresponding authors.
